# Ultrasound findings of pubertal development in girls with cystic fibrosis and their association with clinical outcomes and Tanner staging

**DOI:** 10.20945/2359-3997000000404

**Published:** 2021-09-29

**Authors:** Paula de Souza Dias Lopes, Sandra Helena Machado, Iara Regina Siqueira Lucena, Paulo José Cauduro Marostica

**Affiliations:** 1 Universidade Federal do Rio Grande do Sul Saúde da Criança e do Adolescente Porto Alegre RS Brasil Saúde da Criança e do Adolescente, Universidade Federal do Rio Grande do Sul, Porto Alegre, RS, Brasil; 2 Universidade Federal do Rio Grande do Sul Departamento de Pediatria Porto Alegre RS Brasil Departamento de Pediatria da Universidade Federal do Rio Grande do Sul, Porto Alegre, RS, Brasil; 3 Hospital de Clínicas de Porto Alegre Porto Alegre RS Brasil Hospital de Clínicas de Porto Alegre, Porto Alegre, RS, Brasil; 4 Universidade Federal do Rio Grande do Sul Departamento de Pediatria Porto Alegre RS Brasil Departamento de Pediatria, Universidade Federal do Rio Grande do Sul; Serviço de Pneumologia Pediátrica do Hospital de Clínicas de Porto Alegre, Porto Alegre, RS, Brasil

**Keywords:** Cystic fibrosis; menarche; puberty; ultrasonography, Doppler, Color

## Abstract

**Objective:**

Patients with cystic fibrosis (CF) have a high incidence of pubertal and growth delay. In girls with CF, pubertal delay has an important psychological impact. Still, only a few studies have explored the occurrence of pubertal delay in girls with CF. The aims of this study were to compare the pubertal development of girls with CF compared with healthy controls regarding Tanner staging and pelvic ultrasound and, in girls with CF, correlate the findings with those of spirometry, body mass index, Shwachman-Kulczycki score (SKS), and genotyping.

**Subjects and methods:**

This was a cross-sectional, case-control study including 35 girls with CF aged 6-17 years and following up at the Pediatric Pulmonology Outpatient Clinic of a tertiary hospital. These patients were compared with 59 healthy controls who had undergone pelvic ultrasound as part of another study conducted by the same group. Girls with CF were consecutively enrolled in the study during their annual routine check-up visit. Data collected in the CF group included spirometry and anthropometric results, SKS values, bone age, occurrence of current cystic fibrosis-related diabetes (CFRD) and Pseudomonas aeruginosa colonization, history of meconium ileus, genotype, ultrasound parameters, and Tanner stage.

**Results:**

Pelvic ultrasound findings and Tanner stage reflected less pubertal development in girls with CF compared with healthy controls. Pubertal stage in patients with CF who presented CFRD (3.17 ± 1.16), had chronic colonization by *Pseudomonas aeruginosa* (3.10 ± 1.10), or were homozygous for the F508del mutation (1.91 ± 1.30) was more delayed than in controls (3.41 ± 1.41). Tanner stage correlated with age at menarche, bone age, and anthropometric and ultrasound data.

**Conclusions:**

Girls with CF presented a delay in pubertal development evaluated by Tanner stage and ultrasound parameters, which was more evident in the presence of comorbidities.

## INTRODUCTION

Cystic fibrosis (CF) is a multisystemic genetic disorder mainly associated with nutritional and pulmonary impairment. Patients with CF have a high incidence of pubertal delay and growth deficit; this may be related to malnutrition and severity of lung involvement, which are also prognostic factors for morbidity and mortality in this population ( 1 , 2 ).

Patients with CF experience menarche up to 2 years later than their peers without the disease. Delayed pubertal development causes a significant psychological impact on patients, as it influences self-esteem and body image during a stage of constant life changes. Consequently, these girls tend to participate less in social activities and avoid relationships with the opposite sex (3). This aspect of quality of life in CF is an important topic for investigation, especially considering the dearth of literature on this topic ( 1 ).

Pelvic ultrasound is the most widely used imaging modality to study the genitourinary tract in children and adolescents and may be an important additional method for evaluating puberty ( 4 ). Pelvic ultrasound can detect pubertal changes and is well tolerated by patients because it is simple, readily accessible, and painless. Evaluation with pelvic ultrasound can be performed soon after abdominal ultrasound in girls undergoing yearly check-up. Although physical examination is more accurate to stage pubertal development and detect pubertal delay, pelvic ultrasound is convenient and can be performed by an ultrasound radiologist with whom the girl is already acquainted ( 5 ).

## SUBJECTS AND METHODS

This was a cross-sectional, case-control study. The outcomes of interest were clinical and ultrasound findings of pubertal development in children and adolescents with CF and healthy controls. The sample consisted of 35 girls with CF and 59 healthy controls, aged 6 years to 17 years 11 months and 29 days. The control group was recruited from another study conducted by the same research group ( 6 ).

The inclusion criteria were a diagnosis of CF confirmed by two sweat electrolyte tests performed by the pilocarpine iontophoresis technique (as described by Gibson & Cooke) ( 7 ) or by genotyping; regular follow-up at the Pediatric Pulmonology Outpatient Clinic of *Hospital de Clínicas de Porto Alegre* (HCPA), Rio Grande do Sul, Brazil; and not having had a CF pulmonary exacerbation in the preceding 2 weeks.

The patients were recruited consecutively and exclusively on the day of their annual outpatient check-up visit. Each patient and her parent or legal guardian were approached by the investigator, who provided information on the objectives of the study. Those who accepted the invitation to participate in the study provided written consent. They then completed a questionnaire, designed exclusively for this study, collecting information on the participant’s socioeconomic status, age at diagnosis, genotype, current medications, age of menarche (if menarche had already occurred), history of comorbidities, anthropometric data, and history of chronic colonization by *Pseudomonas aeruginosa* . Colonization by this bacterium was considered chronic when three positive samples were obtained from respiratory secretions (sputum, oropharyngeal swab, or bronchoalveolar lavage fluid) over a 6-month period with an interval of more than 1 month between samples. The patients’ charts were checked to confirm these data, as necessary.

All girls were chaperoned by a legal guardian. Physical examination was done in the presence or absence of the chaperone, as requested by the patient, to maintain privacy.

Breast examination was performed in the anteroposterior and lateral position by one of the two investigators. Some patients were examined by both investigators to verify agreement. For clinical evaluation of pubertal development, we used the Tanner stage based on the breast (B) criterion. Tanner stages are graded on a scale of 1 to 5, in which 1 corresponds to prepuberty and 5 to adulthood ( 8 ).

Weight was measured on a Filizola scale (Filizola, São Paulo, SP, Brazil) with a weighing capacity of 2 to 180 kg and precision of 100 g. Height was measured with a Harpenden stadiometer (Sanny, São Bernardo do Campo, SP, Brazil) with a measuring capacity of 40 to 220 cm and precision of 1 mm. All measurements were performed with the patients barefoot and wearing only a hospital gown. For height measurement, the patients were instructed to stand up straight with their backs leaning as close as possible against the stadiometer, feet together, and head held high at a right angle to the neck, looking directly at a fixed point at eye level. Both measurements, as well as the body mass index (BMI), were normalized by z-scores according to World Health Organization (WHO) curves. The target height was also calculated, and the expected and actual percentiles were determined for each patient, considering their measured height. The target height was estimated by the formula [mother’s height (cm) + father’s height (cm) - 13] / 2. The results of the patients’ annual check-up were recorded, including those of hand and wrist radiographs (for determination of bone age using the method of Greulich & Pyle) ( 9 ) and spirometry (for evaluation of pulmonary function following well-established standardized procedures). Spirometry, forced expiratory volume in the first second (FEV_1_), forced vital capacity (FVC), and FEV_1_/FVC ratio were normalized according to international pulmonary function prediction equations considering the patient’s sex, height, and age ( 10 ). A MasterScreen spirometer (CareFusion, Yorba Linda, CA, USA) was used. All maneuvers were performed with the patient seated and erect, with the head in a neutral position, using a nose clip and a disposable filter mouthpiece ( 10 , 11 ).

The results of complete sequencing of the CF transmembrane conductance regulator ( *CFTR* ) gene in all patients with CF, as well as confirmation of reported comorbidities, were obtained from medical records.

According to the standard HCPA routine, pulmonary function tests and imaging were performed at the time of the annual check-up.

Pelvic ultrasound was added to the routine examination and was performed using a Philips HD11 ultrasound system (Philips Medical Systems, Bothell, WA, USA) equipped with a convex transducer with a frequency of 3-7 MHz. The ultrasound evaluation included color Doppler study of flow through the uterine arteries for analysis of pubertal maturity. All pelvic ultrasound evaluations were performed during routine abdominal ultrasound by the same radiologist with more than 20 years of experience with this modality. Uterine length (cm), transverse diameter (cm), volume (cm^3^), anteroposterior and longitudinal body-to-cervix ratio, endometrial thickness (mm), anteroposterior diameter (cm), ovarian volume (cm^3^), number of follicles smaller than 1 cm, and pulsatility indices (PI) of the right, left, and middle uterine arteries were measured. The PI is the difference between the peak systolic and peak diastolic uterine artery measurements divided by the mean flow velocity ( 6 ).

The sample size was calculated for a statistical power of 80% and a significance level of 5%, considering ultrasound findings in which differences of approximately 0.18 and a standard deviation of 0.27 were found in the uterine body-to-cervix ratio between healthy controls and patients with juvenile idiopathic arthritis (JIA) in a previous study conducted by the same research group ( 6 ). This sample size allows the detection of a minimum relevant value of correlation coefficient between the spirometry/nutritional variables and the ultrasound parameters of 0.5.

Demographic, clinical, laboratory, and imaging data, including ultrasound findings, are presented using descriptive statistics. Student’s *t* test or chi-square test with Yates correction was used for comparison, at a significance level of 5%. Pearson or Spearman correlation coefficient was calculated according to the distribution of the collected data. Logistic regression was used for multivariate analyses. Statistical analyses were carried out using SPSS, version 20.0 (IBM Corp., Armonk, NY, USA).

The study was approved by the CAAE Brazil Platform (49732615.3.0000.5327).

## RESULTS

The sample consisted of 35 female patients with CF, recruited from June 2016 to October 2017, and 59 healthy female controls, all aged 6 to 17 years. All girls who were invited agreed to participate in the study and underwent pelvic ultrasound for evaluation of pubertal maturity. For technical reasons, not all parameters of interest could be visualized in all patients, especially in prepubertal girls.

The mean age of the girls with CF was 11.89 ± 3.61 years, while that of the controls was 12.24 ± 2.92 years. Weight, height, and BMI scores were lower in patients with CF than controls ( Table 1 ).

**Table 1:** Clinical characteristics of the study sample with differences between groups

Variable	Diagnosis	N	Mean (SD)/Median (IR)	p
Age (years)	Controls	59	12.24 (2.92)	0.62
	CF	35	11.89 (3.61 )	
Weight (kg)	Controls	59	52.21 (17.37)	<0.01
	CF	35	38.84 (12.67)	
Height/age z-score	Controls	59	0.86 (0.06-1.66)	<0.01
	CF	35	-0.37 (-0.95-0.21)	
BMI z-score	Controls	59	0.86 (0.43-1.29)	<0.01
	CF	35	-0.11 (-0.51-0.29)	
Target height (cm)	Controls	49	161.75 (157.75-165.00)	0.23
	CF	34	159.50 (156.50-163.75)	
Menarche (years)	Controls	37	12 (10.95-12.00)	0.33
	CF	18	12 (11.00-13.00)	
Tanner staging	Controls	59	4.00 (2.00-5.00)	0.06
	CF	35	3.00 (1.00-4.00)	

CF: cystic fibrosis; SD: standard deviation; IR: interquartile range; BMI: body mass index.

The CF group exhibited differences in relation to the healthy control group regarding several ultrasound parameters consistent with less advanced puberty. Some of the other parameters evaluated were not significantly different, although their mean values indicated greater limitation in pubertal development in patients with CF. No between-group difference in endometrial thickness was observed ( Table 2 ).

**Table 2:** Ultrasonographic characteristics of the study sample with differences between groups

Variable	Diagnosis	N	Mean (SD)/Median (IR)	p
AP body-to-cervix ratio	Controls	58	1.35 (0.27)	<0.01
	CF	35	1.19 (0.29)	
Longitudinal body-to-cervix ratio	Controls	57	1.22 (0.31)	0.01
	CF	35	1.09 (0.19)	
AP left ovary (cm)	Controls	57	1.54 (0.43)	<0.01
	CF	34	1.20 (0.39)	
Volume left ovary (cm^3^)	Controls	57	5.00 (2.95-7.10)	0.01
	CF	34	3.65 (1.27-5.90)	
Longitudinal right ovary (cm)	Controls	58	3.16 (0.75)	<0.01
	CF	34	2.62 (0.68)	
Transverse diameter right ovary (cm)	Controls	58	1.97 (0.58)	<0.01
	CF	34	1.60 (0.50)	
AP right ovary (cm)	Controls	58	1.65 (0.56)	<0.01
	CF	34	1.21 (0.42)	
Volume right ovary (cm^3^)	Controls	58	5.85 (2.97-9.05)	<0.01
	CF	34	2.70 (1.27-4.97)	
Pulsatility indices middle uterine arteries	Controls	55	2.50 (1.90-5.10)	0.13
	CF	25	3.60 (2.45-5.75)	

CF: cystic fibrosis; SD: standard deviation; IR: interquartile range; AP: anteroposterior.

The data presented below refer only to the CF group.

Eleven girls (31.40%) were homozygous for the F508del mutation, and 17 girls (48.57%) were heterozygous for this mutation, which corresponded to 39 alleles. Furthermore, three girls (8.57%) had a 3120+1 G>A mutation in one allele, and two girls (5.71%) had an R1162X mutation in one allele. The remaining girls with CF had one allele different from these.

The mean Tanner stage was 2.86 ± 1.35 in girls with CF and 3.41 ± 1.41 in controls (p = 0.06). Tanner stage was 1 in nine girls (25.71%), 2 in three (8.57%), 3 in eleven (31.42%), 4 in eight (22.85%), and 5 in four (11.42%). In all, 17 (48.57%) girls with CF had not had their menarche yet. All girls older than 13 years presented thelarche. Eleven girls were older than 15 years, and all of them had already had menarche. Still, Tanner stage and anthropometric data were less developed in girls with CF compared with healthy controls. Tanner stage in the control group was 1 in seven girls (11.86%), 2 in eleven (18.64%), 3 in eleven (18.64%), 4 in eleven (18.64%), and 5 in nineteen (32.20%).

All ultrasound parameters, age at menarche, bone age, and anthropometric data correlated significantly with Tanner stage, with correlation coefficients ranging from 0.46 to 0.83. Ultrasound parameters were not significantly associated with pulmonary function ( Supplement 1 ).

Uterine length (r = -0.33, p = 0.04) and volume (r = -0.37, p = 0.02) correlated inversely and weakly with the Shwachman-Kulczycki score (SKS).

Several pelvic ultrasound parameters correlated with BMI z-scores, except for the anteroposterior body-to-cervix ratio, endometrial thickness, number of follicles smaller than 1 cm in both sides, right ovary anteroposterior diameter, right ovary volume, and PI of the right uterine artery in the CF group and longitudinal body-to-cervix ratio, number of follicles smaller than 1 cm in the left ovary, anteroposterior ovary diameter, right ovary volume, and PI of the uterine arteries in the control group. In girls with CF, the mean age at menarche was 11.67 ± 1.37 years and the mean BMI was 18.04 ± 2.18 kg/m^2^, while in the control group, the corresponding values were 11.52 ± 1.23 years and 21.74 ± 4.89 kg/m^2^, respectively (p = 0.69 and p < 0.01, respectively).

**Figure 1:**
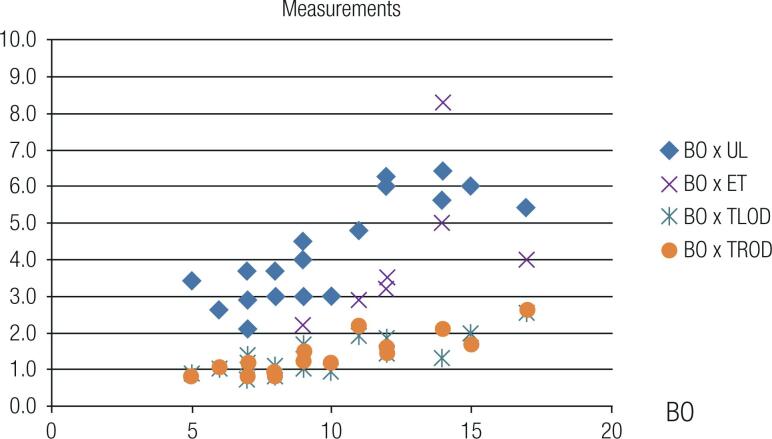
Scatter plot of the distribution of bone age versus pelvic ultrasound measurements in patients with cystic fibrosis. BO: bone age (years); UL: uterine length (cm); ET: endometrial thickness (mm); TLOD: transverse left ovary diameter (cm); TROD: transverse right ovary diameter (cm).

The ultrasound parameters correlated with bone age in years ( Figure 1 ).

Six patients with a diagnosis of cystic fibrosis-related diabetes (CFRD) (17.14%), even after correction for age, had significantly smaller left ovary length (p < 0.01), transverse left ovary diameter (p = 0.02), left ovary volume (p < 0.01), and right ovary length (p < 0.01) than those without this comorbidity. On the other hand, the PI of the right (p = 0.04) and left (p < 0.01) uterine arteries as well as their mean values (p < 0.01) were significantly higher in patients with CFRD, findings consistent with less pubertal maturity.

Ten patients with chronic *Pseudomonas aeruginosa* colonization (28.57%), after correction for age, had left ovary length (p < 0.01), number of follicles smaller than 1 cm in the left ovary (p < 0.01), right ovary length (p = 0.01), transverse right ovary diameter (p = 0.03), and number of follicles smaller than 1 cm in the right ovary (p = 0.04), measurements significantly lower than those in patients without chronic colonization by this bacterium.

Girls with a history of meconium ileus, after correction for age, had a significantly less developed right ovary length (p = 0.04) than those without this comorbidity.

Likewise, patients who were homozygous for the F508del mutation compared with those who were heterozygous for this mutation, after correction for age, had significantly less developed left ovary length (2.20 ± 0.66 cm vs. 3.16 ± 0.82 cm, respectively, p = 0.01), left ovary volume (1.89 ± 1.67 cm vs. 4.90 ± 3.00 cm, p = 0.02), and right ovary length (2.19 cm ± 0.61 vs. 2.95 ± 0.63 cm, p = 0.01).

## DISCUSSION

This novel study found that, compared with healthy controls, girls with CF experience blunted pubertal development assessed using the Tanner criteria and pelvic ultrasound parameters.

Weight, height, and BMI z-scores were lower in patients with CF than in healthy controls. Patients with CF were also shorter, although their target height was similar to that of controls. Compromised nutritional status is a common finding in CF and correlates with FEV_1_, which is a proxy indicator of mortality and is widely used as a pulmonary evaluation outcome measure in studies of CF ( 12 - 14 ). Such differences are relevant since malnutrition is a major risk factor for delayed puberty, not only in patients with CF but also in those with other chronic diseases ( 15 ).

Delayed puberty in the female sex, defined as the absence of thelarche after the age of 13 years, is often found in girls with CF ( 2 ). Both early and delayed puberty are indications for pelvic ultrasound in children and adolescents ( 16 ). However, ultrasonographic assessment of pubertal development is rarely used in clinical practice, and we did not find any publications in which this modality was used in patients with CF. Adequate knowledge of pubertal staging can impact the quality of life of patients with CF, as it allows the development of strategic approaches (especially on the psychosocial front) to address this issue. Furthermore, pubertal development may be a useful indicator of disease severity in patients with CF. Pelvic ultrasound was added to these patients’ evaluation since it was available and because all patients with CF undergo ultrasound evaluation in their annual check-up.

The present study found a correlation between Tanner staging and all ultrasound variables of interest, as well as significant correlations with age, age at menarche, and anthropometric parameters. The onset of puberty may occur as early as the age of 6 years (selected as the lower limit of age for this study), with gradual increases in uterine and ovarian size and gradual reductions in the uterine artery PI; these parameters are detectable on pelvic ultrasound ( 17 ). The PI of the uterine arteries indicates the degree of arterial compliance and resistance to distal flow and is inversely proportional to the size of the uterus and ovaries ( 6 , 16 ).

Many girls with CF included in this study had not yet had their first menstrual period. A Danish study found that, on ultrasound, growth and development of the internal female genitalia in healthy girls precedes the onset of thelarche and puberty ( 18 ), while a German study found that uterine growth begins at the age of 7 years, which provides further evidence of the usefulness of ultrasound as a method for early detection of abnormal pubertal development ( 19 ).

Aligned with our results, a prospective Iranian study found that uterine body length and volume were the parameters that most correlated with age and pubertal stage in healthy girls ( 20 ).

All ultrasound parameters, age at menarche, bone age, and anthropometric data correlated significantly with Tanner stage in our sample. These findings were similar to those of a previous cross-sectional study in girls with JIA ( 6 ). Bone age, a marker of pubertal development, was similarly correlated with the ultrasound parameters of interest.

Endometrial thickness, unlike the other ultrasound variables, was not statistically different between patients with CF and healthy controls. Differences in the phase of the menstrual cycle at the time of measurement may have contributed to this finding since the timing of assessment in relation to the menstrual cycle was not standardized across participants.

Unlike the association of nutritional parameters with some of the ultrasound findings of pubertal maturation found in the current study, there was no association between pulmonary function tests and ultrasound findings, suggesting that pubertal development is impacted more by nutritional status than pulmonary disease ( 21 ). However, it is possible that such an association would be found in a sample of patients with more severe pulmonary involvement than those in the current study. In most cases, patients in the present study did not have severe pulmonary disease, although pulmonary function was sometimes compromised considering their age ( 22 , 23 ).

In the present study, presence of CFRD, history of meconium ileus, and chronic colonization by *Pseudomonas aeruginosa* were associated with delayed pubertal development in patients with CF. Of note, CFRD is strongly associated with pancreatic insufficiency and contributes to worsening pulmonary function, nutritional status, and increased mortality ( 24 ). In addition, insulin may also have gonadotropic activity, modulating the number of FSH or LH receptors. Thus, ovarian cells express insulin receptors, which influence the production of sex steroids. In girls with CF, increases in estradiol and FSH levels occur later, impacting pubertal development ( 25 ). Meconium ileus is the first manifestation of CF in up to 20% of the cases and is associated with pancreatic insufficiency; in the present study, it was also associated with less pubertal development ( 26 ). *Pseudomonas aeruginosa* contributes to a more rapid decline in FEV_1_, more frequent hospitalizations, clinical and radiological deterioration, and risk of death. In short, the comorbidities evaluated in the present study are related both to sexual maturation outcomes and to the systemic severity of CF itself ( 27 ).

Delayed puberty and malnutrition can decrease fertility in patients with CF. The use of CFTR modulators is promising in pubertal delay because of their systemic improvements, *e.g.* , cervical mucus changes leading to increased fertility ( 28 ).

In conclusion, compared with healthy girls, patients with CF exhibit evidence of less pubertal development on pelvic ultrasound. Anthropometric parameters, age, age of menarche, and ultrasound variables all correlated with Tanner staging. Bone age, CFRD, and chronic colonization by *Pseudomonas aeruginosa* correlated with most pelvic ultrasound parameters. It is critical for these comorbidities to be addressed aggressively, given their impact on overall health, including pubertal development.

The evaluation of puberty by pelvic ultrasound in girls with CF is a novel practice and may be beneficial, since early identification of pubertal delay in this population could allow implementation of strategies – such as close follow-up with greater attention to psychosocial issues – to improve quality of life.

## Appendix

**Supplement 1:** Correlation coefficients between Tanner staging and clinical or ultrasonographic variables in patients with cystic fibrosis

Variable	N	R	P
Age	35	0.83	<0.01
Age at menarche	35	-0.78	<0.01
Uterine length	35	0.78	<0.01
Uterine transversal	35	0.72	<0.01
Uterine AP	35	0.71	<0.01
Uterine volume	35	0.75	<0.01
AP body-to-cervix ratio	35	0.64	<0.01
Longitudinal body-to-cervix ratio	35	0.62	<0.01
Endometrial thickness	25	0.46	0.02
Left ovary longitudinal size	34	0.72	<0.01
Transversal left ovary	34	0.82	<0.01
AP left ovary	34	0.59	<0.01
Left ovary volume	34	0.79	<0.01
Follicles number < 1 cm in left ovary	31	0.48	<0.01
Right ovary longitudinal size	34	0.56	<0.01
Transversal right ovary	34	0.59	<0.01
Right ovary AP	34	0.61	<0.01
Right ovary volume	34	0.62	<0.01
Follicles number < 1 cm right ovary	32	0.46	<0.01
Right uterine artery pulsatility index	29	-0.37	0.04
Left uterine artery pulsatility index	27	-0.54	<0.01
Mean uterine arteries pulsatility index	25	-0.56	<0.01
Bone age (years)	18	0.82	<0.01
Bone age (z-score)	35	0.40	0.01
Forced expiratory volume in 1 second (FEV1; z-score)	35	0.10	0.53
Forced vital capacity (FVC; z-score)	35	0.08	0.62
FEV_1_/FVC ratio	35	0.10	0.56
Shwachman-Kulczycki’s score	35	-0.08	0.61
Height	35	0.83	<0.01
Weight	35	0.90	<0.01
Body mass index (BMI)	35	0.75	<0.01
Height/age z-score	35	0.36	0.03

R: correlation coefficient; AP: anteroposterior; FEV1/FVC: Tiffeneau index.
